# Differential Genetic Susceptibility to Child Risk at Birth in Predicting Observed Maternal Behavior

**DOI:** 10.1371/journal.pone.0019765

**Published:** 2011-05-16

**Authors:** Keren Fortuna, Marinus H. van IJzendoorn, David Mankuta, Marsha Kaitz, Reut Avinun, Richard P. Ebstein, Ariel Knafo

**Affiliations:** 1 Department of Psychology, the Hebrew University of Jerusalem, Jerusalem, Israel; 2 Centre for Child and Family Studies, Leiden University, Leiden, The Netherlands; 3 Department of Obstetrics and Gynecology, Hadassah University Hospital, Jerusalem, Israel; 4 Department of Neurobiology, the Hebrew University of Jerusalem, Jerusalem, Israel; 5 Department of Psychology, National University of Singapore, Singapore; VIB & Katholieke Universiteit Leuven, Belgium

## Abstract

This study examined parenting as a function of child medical risks at birth and parental genotype (dopamine D4 receptor; DRD4). Our hypothesis was that the relation between child risks and later maternal sensitivity would depend on the presence/absence of a genetic variant in the mothers, thus revealing a gene by environment interaction (GXE). Risk at birth was defined by combining risk indices of children's gestational age at birth, birth weight, and admission to the neonatal intensive care unit. The DRD4-III 7-repeat allele was chosen as a relevant genotype as it was recently shown to moderate the effect of environmental stress on parental sensitivity. Mothers of 104 twin pairs provided DNA samples and were observed with their children in a laboratory play session when the children were 3.5 years old. Results indicate that higher levels of risk at birth were associated with less sensitive parenting only among mothers carrying the 7-repeat allele, but not among mothers carrying shorter alleles. Moreover, mothers who are carriers of the 7-repeat allele and whose children scored low on the risk index were observed to have the highest levels of sensitivity. These findings provide evidence for the interactive effects of genes and environment (in this study, children born at higher risk) on parenting, and are consistent with a genetic differential susceptibility model of parenting by demonstrating that some parents are inherently more susceptible to environmental influences, both good and bad, than are others.

## Introduction

A great deal of research has substantiated the critical role of parenting in children's development and functioning early on as well as later in life. Parental quality of care (e.g., maternal sensitivity) is predictive of a variety of child outcomes, such as attachment security [1], [2], social understanding and behavior [3], and relationship quality (e.g., with siblings [4]). It is therefore important to continually explore determinants of parenting in order to better understand why parents parent the way they do [5]. Parenting is a multifaceted behavior, influenced by a multitude of factors including the parent's own characteristics, the child's contributions, the family context, and beyond. More critically, parenting is a dynamic process in which the various influences constantly interact to shape moment-to-moment parent-child interactions. Identification of such factors and processes is imperative in explaining the variability in parenting behavior across individuals and contexts. In this study we integrate a focus on the effects of both child-related risk and parents' genotype on parenting as to identify transactional processes taking place between parents' genetic tendencies and the challenges they face when it comes to parents' abilities to respond sensitively to their children.

Risk was defined in terms of child medical risk at birth, indexed by children's gestational age at birth, birth weight, and a length of stay at the neonatal intensive care unit (NICU). The (parent) gene that we focused on is the dopamine D4 receptor (DRD4). Several studies have shown that the presence of the exon III 7-repeat allele on the dopamine gene is related to the differential susceptibility of children to parental influences. More recently it has been shown to moderate environmental and child-related stress on parents' sensitivity to their children [6], [7].

### Risk at Birth and Parenting

Having a preterm or low birth-weight infant represents a major stressor for most parents. There is evidence that mothers of preterm infants have more immediate psychological distress and stress related to parenting than mothers of term infants [8], especially if the newborn is at higher risk due to very low birth weight (VLBW; <1500 g) and is at significant medical risk due to additional medical complications (e.g., Intracerebral hemorrhage) and expected developmental delays [9]. Mothers of preterm, VLBW infants, report being anxious about many aspects of their children's health, developmental prognosis, care, and future. Parental anxiety has been shown to persist over a period of a few months, well after the critical neonatal period had passed [10]. Additional longitudinal findings indicate continued effects of VLBW and duration of stay at the NICU on parenting stress during the first three years of the child's life [11], and even into the child's sixth year [12].

Continued high maternal anxiety can be thought, and was indeed found, to adversely affect mothers' ability to interact sensitively with their infants [10], [13]. Some studies report mothers of preterm infants to be less responsive to infants' cues than mothers of term infants, at times becoming overactive in effort to stimulate the child [14], [15]. The immature behavior of the preterm infant has been cited as another reason for differences in maternal behavior. VLBW infants are described as behaviorally challenging. In comparison to full-term infants, preterm infants are less able to handle stimulation as they are unable to control attentional states, and tend to become over-aroused, disorganized and distressed when stimulated [16]. Mothers of VLBW children tend to perceive them to be more stressful, demanding, distractible, hyperactive, and less acceptable and adaptable compared with the mothers of term children [9], [17]. Thus, mothers of VLBW children face the demanding task of attempting to care for an infant whose behavioral cues are often difficult to interpret [18], [19], making it more difficult to engage in positive, sensitive interactions with their child.

All that said, empirical findings of the association between preterm birth/birth weight and parenting stress/mother-child interactive behavior are mixed [10]. Many studies find preterm birth and low birth weight (not accompanied by other complications) to be unrelated to maternal stress [9], [17], [18] nor to maternal behavior during mother-child interactions [10]. Thus, birth risks may be associated with less sensitive parenting for some parents but not others. This unexplained variability can be due to additional factors not accounted for in those studies. Recent findings, reviewed next, point to genetic moderation of environmental stressors on parenting, suggesting the need to investigate the role of mothers' genotype.

### Genetic Susceptibility of Parenting

Although parenting has been widely researched with regards to the experiences and attributes that affect parenting, few studies of genetic influences on parenting have been conducted in humans [20], [21]. Only very recently molecular genetic studies have emerged that investigate the effects of parents' genes, in combination with their experiences, on their parental behavior [6], [7], [22]. This gene by environment interaction (GXE) research, a newly developing avenue of parenting research and a relatively novel approach in general, compares the association between an environmental variable and a phenotype in individuals with different genetic profiles. Although the theoretical importance of GXE has long been known [23], strong empirical evidence in psychological research has emerged only in the last decade. Parenting GXE research draws on child development molecular genetic findings that point to some children being more influenced by their rearing conditions than others as a function of the presence (vs. absence) of specific genetic variants [24], [25].

Originally such individuals believed to carry a certain risk—whether genetic, behavioral (e.g., negative emotionality [26], [27]) or physiological [28] in nature—were considered to be “vulnerable” [29]- that is, at high-risk for problematic developmental outcomes when experiencing adversities, due to this dual-risk situation [30]. Indeed, early findings fitted a *Diathesis-Stress model* [31], [32], in which individuals who exhibit some inherent risk (i.e., diathesis) and are exposed to harsh conditions (i.e., stress; e.g., maltreatment) are disproportionately or even exclusively likely to manifest a psychopathological condition.

More recently, Belsky [33], [34] and colleagues [35]–[38] have proposed an alternative pattern of GXE, that of *Differential Susceptibility*. Differential susceptibility theory points to the role of the individual characteristics in moderating not only the effects of stressful environmental conditions but also of supportive contexts, on human development. Thus, this view extends the traditional diathesis-stress model, by making the observation that individuals disproportionately vulnerable to adversity are also most likely to benefit from highly supportive environments (see also [39]). Consistent with this view, it is becoming increasingly evident that individuals differ in their susceptibility to the environment, both negative and positive, such that susceptible (malleable) individuals are more strongly affected for better *and* for worse [35], [36].

Differential susceptibility theory emphasizes genetic influences on human plasticity. Indeed, empirical evidence for genetic moderation of environmental effects in line with this view is beginning to accumulate, and specific “susceptibility genes” have been identified, especially those which regulate the serotonin and dopamine brain systems. Specific to the present investigation, we focus on findings involving a polymorphism of the dopamine D4 receptor gene, the DRD4-III 48 bp repeat, which has two to eleven repeats (4 and 7 being the most common in Caucasian samples; [40]). The DRD4-III 7-repeat allele (DRD4-7R) has been highlighted as a susceptibility gene in many GXE studies. It was found to moderate associations between parenting and a variety of child outcomes such as disorganized attachment, externalizing problems, and prosocial behavior [41]–[43]. For example, in an experimental intervention of promoting positive parenting and sensitive discipline, child behavior problems subsequently improved only in the group of children carrying the DRD4-7R allele [44]. These powerful findings suggest that this particular allele (i.e., variant) of the gene heightens susceptibility to risky as well as supportive environments, at least in children.

GXE findings are also evident in studies on adults, mostly focusing on psychiatric problems as the outcome [45], [46]. Despite evidence for the heritability of parenting [21], very few studies have been conducted on the specific genes related to parental behavior, and even fewer on GXE effects. However, based on previous findings of genetic susceptibility in children and adults there is reason to expect that similar processes take place to influence parenting quality [47]. More specifically, it is hypothesized that susceptibility genes, perhaps the very same ones identified in children, act to moderate the influence of life experiences on adults' abilities to care for their offsprings. Evidence that parenting behavior can be predicted by GXE would further our understanding concerning individual differences in parenting quality, so critical for children's development and family functioning. Such susceptibility to positive influences may also explain why some parents, but not all, benefit from experimental interventions designed to improve parenting ([44], see discussion in [38]).

In the first study to test this expectation [6], the association between daily hassles (i.e., stressful life events) and sensitive mothering was found to be moderated by a combination of the DRD4-7R and another dopamine-related gene (COMT) in a “for better and for worse” manner. In other words, mothers carrying this gene combination were less sensitive to their children when confronting high levels of daily hassles and more sensitive when experiencing fewer hassles. Another study [7] provided further support for GXE interactions involving the DRD4-7R allele operating in parents; infant fussy-difficult temperament was associated with maternal sensitivity only for mothers who carry the DRD4-7R allele. This evidence for the involvement of DRD4-7R GXE interaction regarding parenting calls for an investigation of its role with regards to neonatal medical risk at birth in predicting levels of maternal sensitivity.

### The Current Study

In this study we tested the role of maternal DRD4 genotypic variation in susceptibility to children's medical risk at birth and observed parenting behavior towards those children at age 3.5 years. Participants were a sub-sample of a larger study of mothers and their twins [48]. It is worth noting that risks involved in pregnancy and birth are exacerbated when it comes to twins. The news of having twins is often followed by a somewhat stressful prenatal period, as any twin pregnancy is considered a high-risk pregnancy. Preterm delivery rates among twins are significantly higher than those of singletons (average gestational age at twin delivery is 36 weeks), and birth weight is significantly lower [49].

One of our goals was to test whether DRD4-7R acts as a diathesis to stress or more generally as a susceptibility marker to both high and low stress. In order to identify individuals who not only are negatively affected by adverse conditions but also benefit most from positive experiences, it is necessary to include the entire range of the environmental factor under investigation, which can be the absence of the stressor [37]. Therefore, both high and low birth risk was considered.

A possibility we wished to account for is that risk at birth is confounded with children's health problems, and thus that it is not risk at birth, but rather later health problems, that are moderated by mothers' genotype to predict parenting. Preterm infants are at increased risk for persistent medical difficulties across developmental domains [19]. We have, therefore, also controlled for children's hospitalizations in the analyses.

## Methods

### Ethics Statement

Ethics approval for this study was obtained from the ethics committee at Herzog Hospital Jerusalem and by the Israeli Ministry of Health higher committee on human medical research. Mothers provided written informed consent before enrolling in the study.

### Participants

Participants were mothers and their twins taking part in a longitudinal study of twins [48], examining genetic and socialization influences on development. When the twins reached the age of 3, mothers were asked (via mailed questionnaires) about the course of the twins' pregnancy, delivery, the postnatal period, and the twins' health problems, as well as additional information beyond the scope of this report. By the time of this report, 187 families (the mother and her two children) visited our lab when the twins were about 3.5 years of age (*M* = 44.25 months, *SD* = 2.95). Visits were scheduled at a time when children were likely to be at their best and typically completed in less than 2 hours. During the session, the twins were observed individually in a series of tasks. Mothers were then observed interacting separately with each twin, and provided DNA samples.

The current sample (*N* = 104 mothers, 199 twins) consisted of families for which (a) mother-child interactions were available with at least one twin (in 14 families mother-child interactions were either not conducted or could not be coded due to technical problems, and in nine families interactions were available with one of the twins only), and (b) mother's DNA was available (68 mothers declined giving samples, and in four cases the quality of the sample did not enable analysis). There were no significant differences between the current sample and the mothers for whom DNA or behavioral assessment were unavailable on any of the study variables (i.e., birth-risks, maternal behaviors, hospitalizations), maternal education, socioeconomic status, and child age, though mothers in the former were somewhat younger (*M* = 33.68, *SD* = 5.05) than the latter (*M* = 35.32, *SD* = 6.18; *t*(174) = 1.93, *p*<.06, *d* = .29). On average mothers had completed 15.54 years (*SD* = 2.57) of formal education. Twins' zygosity was determined by DNA samples. The children consisted of 34 monozygotic (MZ) twin pairs (17 female pairs, 17 male pairs), and 70 dizygotic (DZ) twin pairs (18 female pairs, 25 male pairs, 27 mix-sex dyads).

### Measures

#### Risk at birth

This composite reflects the degree of the twins' medical risk at birth, indexed by gestational age at birth, birth weight for gestational age, and length of stay at the NICU (adjusted for gestational age), defined as follows:

#### Birth week risk

Delivery at gestational age of 24 to 32 weeks was considered high risk (assigned a score of 2; 9%), delivery at 33 to 36 weeks was rated as moderate risk [50] (score of 1; 35%), and birth at 37 weeks or more was assigned a low risk score [51] (score of 0; 56%).

#### Birth weight risk

High birth weight risk (score of 1; 15%) was assigned based on the child's low birth weight relative to twins of the same gestational age at the time of birth, defined as falling below the 10^th^ percentile relative to Israeli population norms for twins [49]. All others were assigned a low risk score (0; 85%).

#### NICU risk

Admission to the NICU was considered a risk index based on the following criteria [52]: Twins who were born at 32 weeks of pregnancy or less and stayed for over 4 weeks at the NICU were considered high risk. Children who were born at 33 weeks or more and had to be admitted to the NICU for any length of time were also judged as high risk (score of 2; 19%). If the children were born at 32 weeks or less, a stay of under 4 weeks at the NICU was assigned to be moderate risk (1; 7%). Finally, no admission to the NICU after birth was coded as low risk (0; 74%).

A principal component analysis indicated that week, weight, and NICU risks loaded on one factor accounting for 55.2% of the variance (loadings ranging from .40 to .89). Therefore, total risk at birth was the factor score based on the three (standardized) risk scores. In addition, since the focus of this paper is on mothers as the unit of analysis, and given high inter-pair twin correlations on birth weight (*r* = .77, *p*<.001 for MZ pairs, *r* = .73, *p*<.001 for DZ pairs) and admission to the NICU (90% were both either admitted to the NICU or not, and for only 10% one twin was admitted while the other not) birth risk scores were averaged across twins within pairs.

#### Children's hospitalizations

Mothers were asked whether one of the twins or both were ever hospitalized (yes/no), and when (open-ended). Answers were screened to exclude NICU hospitalizations, and were coded as 1 if one or both of the twins within a pair were hospitalized and 0 if neither of them were ever hospitalized. Children's hospitalizations were associated with risk at birth scores, *t*(99) = 2.44, *p*<.05, *d* = .49).

#### Observed maternal behavior

Mothers were observed during 10 minutes of free-play with each of their twins. The mother entered each room to play with one of her twins while the other twin was kept busy by the examiner in a separate room. The same procedure was later repeated with the other twin (there were no systematic effects regarding which twin the mother played with first). A colorful set of play-dough and modeling tools was provided and the mothers were asked to play with their child as they would normally do. Mothers' interactions with the children were digitally recorded and later scored by trained research assistants. Observers rated maternal behaviors for each 2-minute segment, and then scores were averaged across the 10-minute session. Different observers independently rated mothers' behaviors towards the two children within each twin pair.

*Maternal Sensitivity* was conceptualized as a multidimensional construct that included maternal warmth, autonomy support, and responsiveness. Mother's *Warmth* was rated according to the mother's expressed positive affect towards the child, physically and verbally (e.g., smiles, hugs, affectionate looks, comments directed at the child expressing joy in the interactions). Ratings ranged from 0 (no expressions of positive affect) to 4 (frequent, repeated expressions of positive affect throughout the interaction). *Autonomy Support* was defined as parenting behavior that enhances a child's sense of value and personal control, shows respect for child's ideas, supplies feedback, offers choice, and acknowledges child's cues [53]. The scale ranged from 0 (no signs of autonomy support: mother rarely offered the child choice, adapted to child needs, or provided feedback) to 4 (strong and consistent provision of autonomy support). Maternal *Positive Responsiveness* reflects the frequency in which the mother responded to the child's needs (e.g., child's distress, child's bid for maternal attention, child's need for instrumental help) in a prompt, contingent, supportive, genuinely interested, empathic manner (“exceptional responsiveness” from [54]). Since only maternal behaviors that occurred in response to the child's behavior were rated on this scale, we controlled for the variability in the number of child-elicited events across dyads by dividing the number of positive responses by the total number of child-elicited events, resulting in a percentage of positive responsiveness out of all possible types of responses. A factor analysis revealed that the mothers' warmth, autonomy support, and responsiveness loaded on one factor (.86, .86, and.74, respectively), and accounted for 67 percent of the variance. On this basis, we averaged the standardized scores of these measures to derive a factor of overall maternal sensitivity.

*Maternal Negativity* included maternal negative affect, such as angry or hostile tone and facial expressions, expression of impatience or boredom, as well as verbal comments of dissatisfaction and criticism of the child. The scale ranged from 0 (little or no negative affect) to 4 (consistent, strong negative affect).

As the focus was on the mothers' responses toward both their twins, and the between-twin correlations for maternal sensitivity and negativity indicated similarity in maternal behavior across twins (sensitivity: *r* = .47, *p*<.001; negativity: *r* = .26, *p*<.01), ratings were averaged within pairs. The intraclass consistency coefficient of maternal behaviors between the coders across the five 2-minute segments based on 63 reliability cases were .83 for sensitivity and .87 for negativity.

#### DRD4-III polymorphism

DNA was extracted from 20 ml of mouthwash samples using the Master Pure kit (Epicentre,Madison,WI. PCR amplification was carried. The exon III repeat region of the DRD4 receptor was characterized using PCR amplification procedure (using a Reddy Mix kit, AB gene, Surrey UK) with the following primers: F5′-TTCCTACCCTGCCCGCTCATGCTGCTGCTCATCTGG-3′; R5′-ACCACCACCGGCAGGACCCTCATGGCCTTGCGCTC-3′. PCR reactions were performed using 5 µl Master Mix (Thermo scientific), 2 µl primers (0.5 µM), 0.6 µl Mg/Cl_2_ (2.5 mM), 0.4 µl DMSO 5% and 1 µl of water to total of 9 µl total volume and an additional 1 µl of genomic DNA was added to the mixture. All PCR reactions were employed on a Biometra T1 Thermocycler (Biometra, Güttingem, Germany). PCR reaction conditions were as follows: preheating step at 94.0°C for 5 min, 34 cycles of denaturation at 94.0°C for 30 s, reannealing at 55°C for 30 s and extension at 72°C for 90 s. The reaction proceeded to a hold at 72°C for 5 min. The mixture was then electrophoresed on a 3% agarose gel (AMRESCO) with ethidium bromide to screen for genotypes.

Twenty eight percent of the mothers were carriers of the 7-repeat allele (three homozygous carriers), and 72% were not carriers (of which 77% were homozygous for the 4-reapeat allele), a distribution similar to that found in a comparable sample of mothers [7; 34% carriers]. Genotypes were in Hardy-Weinberg equilibrium, *χ* ^2^(1) = .16, *ns*, reflecting a random combination and stable frequency of the DRD4 repeat alleles in the population.

## Results

### Descriptives and Bivariate Correlations

Table 1 provides the means and standard deviations of the study variables by the presence or absence of maternal DRD4-7R allele. There were no significant differences between mother carriers and non-carriers of the 7R allele on risk at birth scores, hospitalizations, or ratings of maternal behavior. We next examined the bivariate correlations between risk at birth and mothers' parenting in the presence and absence of the mothers' DRD4-7R allele (Table 2). Risk at birth was not associated with maternal behaviors in the full sample. Likewise, among 7-absent mothers, no relation was found between risk and maternal sensitivity indicators/negativity. In contrast, among 7-present mothers a significant negative correlation was found between risk at birth and each of the maternal sensitivity scales, as well as the sensitivity composite score. In fact, risk at birth accounted for 19% of the variance in maternal sensitivity among the 7-present mothers. The correlations with maternal negativity were in the expected direction —risk was uncorrelated with negativity for the 7-absent mothers and higher risk was correlated with stronger negativity for the 7-present mothers—but this was only a marginally significant effect (*p*<.10). Further analyses showed identical results when children's hospitalizations were controlled (i.e., regressing risk at birth on maternal outcomes while controlling for hospitalizations).

**Table 1:** Means and Standard Deviations of Study Variables by the Presence and Absence of Maternal DRD4-III 7-Repeat Allele.

	7-absent		7-present	
	*M*	*SD*	*M*	*SD*
Risk at birth	.02	1.01	−.06	.98
Overall maternal sensitivity	−.02	.67	.03	.65
Maternal warmth	2.45	.55	2.48	.56
Maternal autonomy support	2.41	.54	2.58	.61
Maternal responsiveness	5.24	5.14	4.24	4.29
Maternal negativity	.34	.36	.36	.30
Children's hospitalizations	.41	.49	.30	.47

**Table 2:** Correlations between Risk at Birth and Maternal Behavior as a Function of the Presence/Absence of Maternal DRD4-III 7-Repeat Allele.

	Autonomy Support	Warmth	Responsiveness	Overall Sensitivity	Negativity
All mothers	−.09	−.09	−.04	−.09	.02
7-absent	.03	.01	.06	.04	−.05
7-present	−.35*	−.35*	−.39*	−.44**	.25

*p<.05;

**p<.01, 1-tailed.

### Regression Analysis Predicting Observed Maternal Behavior

As a formal test of the hypothesis that the variation in the DRD4 gene moderates the association between risk at birth and maternal behavior, we regressed mothers' observed sensitivity on risk at birth and their DRD4-III polymorphism (both centered, 7-present was coded 1, and 7-absent −1), as well as the centered interaction term. There were no main effects of either risk at birth (β = −.09, *t* = −.94, *ns*) or presence of the DRD4-7R allele (β = .03, *t* = .26, *ns*). However, the interaction between risk and DRD4 significantly predicted maternal sensitivity (β = −.21, *t* = −2.18, *p*<.04), accounting for 5% of the variance, thus indicating that the relation between risk at birth and later maternal sensitivity depended on the variation in the DRD4 gene. The results remained unchanged with children's hospitalizations statistically controlled in the regression model.

A similar regression model was used to predict maternal negativity. Again, there were no main effects for risk (β = .03, *t* = .21, *ns*) and DRD4 (β = .03, *t* = .32, *ns*), however the effect of the interaction term in predicting negativity did not reach significance either (β = .12, *t* = 1.21, *ns*). This lack of a significant GXE effect may be due to insufficient variation in negativity as observed in the context of the free-play task, which is not especially designed to evoke it. To test this possibility, we compared the variance of observed negativity to that of maternal warmth and autonomy support (which were all rated on the same 0–4 scale). Mauchly's sphericity test (testing for the equality of variances of repeated measures) indicated that the variance of the maternal negativity scale was indeed significantly lower than that of the other scales, χ^2^ = 60.09, *p*<.001.

**Figure 1:**
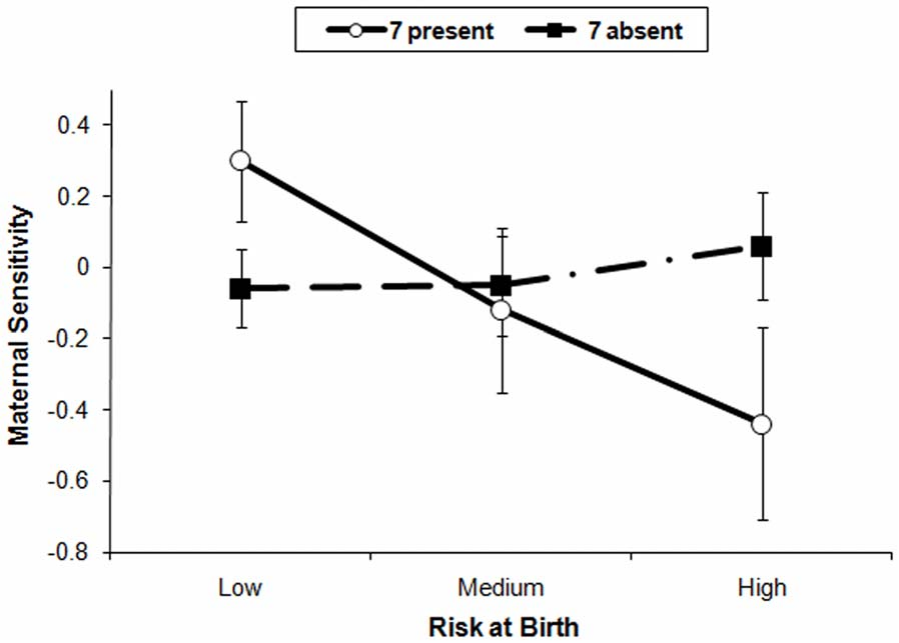
Mean maternal sensitivity based on risk at birth and the presence/absence of maternal DRD4-III 7-repeat allele. Mean levels (and standard errors) of observed maternal sensitivity as a function of child risk at birth (low, medium, and high) and the presence or absence of maternal DRD4-7R allele. Mothers who are non-carriers of the 7 allele showed no difference in levels of sensitivity under conditions of high, medium, and low risk at birth. Mothers who are carriers of the 7 allele were more sensitive to low-risk children and less sensitive to high-risk children.

The significant interaction predicting maternal sensitivity was further probed in order to fully understand the combined effect of DRD4 and medical risk at birth in predicting maternal sensitivity. Of particular interest was to determine whether the presence of the DRD4-7R allele acts only as a vulnerability to an adverse situation (in this case, having a high-risk child birth), which would be consistent with a diathesis-stress model, or whether it relates to a susceptibility to both negative as well as positive contexts, which would be consistent with a differential susceptibility model. To test this we needed to depict a full range of this environmental variation. Although twins' gestation is considered a higher risk pregnancy and birth in general in the obstetrics literature, for descriptive purposes we divided risk at birth into three subcategories consisting of low risk (those falling under .50 standard deviations below the mean, 47%), medium risk (29%), and high risk (.50 standard deviations above the mean, 24%). Figure 1 depicts mean levels (and standard errors) of observed maternal sensitivity as a function of risk level (low, medium, and high) and the presence or absence of the DRD4-7R allele in the mothers. As the graph illustrates, mothers who are non-carriers of the 7 allele showed no difference in levels of sensitivity under conditions of high, medium, and low risk at birth. In contrast, mothers who are carriers of the 7 allele were more sensitive to low-risk children and less sensitive to high-risk children, supporting a differential susceptibility model.

### Individual Twins Follow-Up Analyses

In addition to examining the associations between scores averaged across twins, in follow up analyses we also tested whether the effects replicate for the individual twins (*N* = 199) when their scores (risk at birth and maternal sensitivity) are treated separately. Because twin pairs come from the same family their scores cannot be assumed to be independent. To account for this nonindependence among twins, generalized estimating equations with robust covariance estimators were used with twin pairs clustered by family. Results were similar to those reported above. No significant relationship was found between risk at birth and maternal sensitivity for the full sample (B = −.08), Wald χ^2^ (1) = 1.46, ns, as well as among the DRD4-7R non-carrier mothers only (B = −.004), Wald χ^2^ (1) = .003, ns. In contrast, among the mothers carrying the 7R allele, risk at birth was negatively associated with maternal sensitivity (B = −.31), Wald χ^2^ (1) = 6.27, *p* = .01.

## Discussion

A broad literature attests to the importance of sensitive parenting for optimal development of children. Identification of factors that can interfere with sensitive mothering is a primary goal in the study of infant and child mental health. Among relevant studies some have shown that preterm birth and child's low birth weight are associated with parenting stress and less sensitive mother-child interactive behavior for some parents. A high-risk birth of twins may especially challenge parents' abilities to sensitively care for their children [10]. Importantly, these associations are not seen consistently for all parents within and across studies. In this study we did not find support for a direct association between risk at birth and parenting. Rather, we demonstrate that the parenting of some mothers, those carrying the DRD4-7R allele, appears to be vulnerable to the stress associated with high neonatal medical risk over time, yet for mothers who do not have this particular allelic expression risk at birth was not related to sensitivity.

In addition, those same “vulnerable” mothers were also more likely to respond most sensitively to their children if twins were born at lower risk. In keeping with differential susceptibility evaluation criteria, as the environmental range covered both ends of the risk at birth spectrum, we saw a cross-over interaction: mothers carrying the 7R allele showed very low abilities to respond sensitively to their child following high medical risk, whereas mothers of low risk children showed high levels of sensitivity; higher, in fact, than non-carrier mothers under similar circumstances. As such, these findings are consistent with a differential susceptibility model [33], [34] predicting that the very same characteristics that make individuals disproportionately vulnerable to adversity also make them more likely to benefit from favorable contexts.

Some of the unknowns in differential susceptibility theory are the mechanisms responsible for translating the variation in certain genes into differential plasticity. We, as well as others [6], [7], have shown that differences in the DRD4 gene are related to sensitive parenting (depending on stress levels). Being a sensitive parent depends on accurately reading the child's signals and responding adequately and promptly [1], a task requiring motivation, focused attention, accurate interpretation of the child's signals, and flexibility in responding. It has been suggested that DRD4 is associated with responsivity to reward and punishment through its effect on dopamine-related neural circuits regulating attentional, motivational, and reward mechanisms [44], all of which seem to be involved in sensitive parenting. The 7R allele, in particular, is associated with lower dopamine reception efficiency [55], and less adequate behavioral regulation such as impulsive behavior, ADHD, and high novelty seeking behavior, whereas the short variants of the gene are related to rigidity and inhibition [55], [56]. It is thus suggested that the presence of the 7R allele makes individuals more distractible by less relevant stimuli (e.g., stress-related and not related to the child's signals) while they are simultaneously less able to regulate stress and therefore more susceptible to its influences. On the positive side, carriers of the 7R allele might also be more attentive and open to the child's signals if other, interfering stimuli originating from a stressful environment are lacking. While studies of the DRD4-III polymorphism at the cellular level are imperative in order to facilitate our understanding of parental susceptibility to stress, studies about how variations in this gene influence brain activation are ongoing (e.g., [57]) and will provide additional insights into the processes involved.

As a direction for future study to further dissect the influences of the factors involved in shaping maternal behavior, it would be of interest, in addition to focusing on maternal genotype, to assess the contribution of *child* genotype and behavior to maternal behavior (a gene-environment correlation). Family influences are not unilateral and children affect the parenting they receive. Thus, further research might point to ways in which children's DRD4 genotype interplays with maternal DRD4 and environmental factors to predict parental behavior.

The study has several strengths. Foremost, it is among the first studies to implement the field of molecular genetics in studying parenting behavior by taking into consideration the effects of parental genes, in combination with child-based environmental influences, to study the quality of parent-child interactions. Second, differential susceptibility theorists as well as molecular genetic scholars call for inclusion of observed behavioral assessments (or other valid measures of the phenotype of interest [55]). In this study, parenting behaviors were directly observed and rated according to validated protocols. Importantly, the environmental stressor was an objective measure of medical risk at birth. That is, in contrast to the two previous studies on differential susceptibility of parenting [6], [7], we did not rely on subjective reports of the environment, which may suffer from reporter biases and other limitations. Also, although the putative diathesis was assessed just a few months before the outcome, given its content (birth week, birth weight, and length of stay at the NICU) it could not have been affected by later experiences. Finally, the use of very different measures (DNA, behavioral observations, and medical history), reduced the likelihood of shared method variance.

The study also has some limitations. First, the limited sample size prevented analysis of additional genetic polymorphisms. The converging evidence across studies of children and adults makes a strong case for DRD4-III as a differential susceptibility gene. DRD4 might serve as a powerful index of an underlying dopamine-related set of genes affecting the parental phenotype in interaction with a specific environment. Nevertheless, differential susceptibility may be more adequately accounted for by multiple genes, proposed to have cumulative effects [38], [55]. Gene-gene interactions and epigenetic changes [58] may also account for susceptibility and are important to include in future genetic studies of parenting.

Second, in order to test a fit to the differential susceptibility model we included an environmental influence on parenting varying from highly stressful (having a newborn at medical risk) to a low stress condition (delivering a baby at no immediate risk). This variation did prove diverse enough to identify mothers who appear more susceptible to both the high and low ends, and were observed to have both lower and higher levels of sensitivity, respectively. However, to truly demonstrate susceptibility to enriched, highly positive environments, it is recommended to not merely consider the presence versus absence of adversity but also focus on intrinsically positive life experiences or favorable environments [37], [41]. More direct evidence for positive outcomes for susceptible parents in favorable child-rearing contexts is recommended in future research.

Third, the birth-risk index used in the study is limited in that it does not directly assess specific medical risks and complications (e.g., intracerebral hemorrhage) that are not necessarily associated with preterm, low birth weight, and NICU hospitalization. Additionally, we did not address the mediating mechanism between child risk at birth and maternal sensitivity three and a half years later that can explain what mothers are responding to in their high versus low-birth-risk children resulting in different levels of sensitivity. As detailed in the introduction, it has been shown that children who were at high risk at birth can be more stressful for (susceptible) mothers than are children who were born at low risk, who may provide a more ‘beneficial’ environment for their mothers. The current report does not address this issue, but future studies can contribute further by identifying child and mother -related mechanisms (e.g., child emotional regulation, maternal anxiety) that mediate this association.

Forth, maternal behavior was assessed during a single observation of mother-child interaction. As responses to one's child vary across contexts, a more extensive assessment of parenting employing repeated observations would increase the confidence that the observed behaviors reflect mothers' interactive patterns. For example, it appears that the observational context employed in this study (i.e., free play) did not evoke much variation in maternal negativity. That said, confined to a single observation we chose a relatively unstructured task (yet constant across mothers in terms of the instructions and material provided) as to minimize constraints on maternal behavior, and thus to allow for individual differences in sensitivity to emerge.

Finally, our sample consisted of mothers of twins. On one hand, it likely assisted in having enough variance on the risk measure, as twins are more likely to be preterm and of low birth weight. On the other hand, the unique challenges in parenting twins may limit the generalizability of our findings. Twins' mothers may be at greater risk for increased anxiety [10]. Also, as twin pregnancy and birth are expected to have some complications, having twins who are full term, healthy newborns may actually be considered a highly positive experience, whereas in singletons it is assumed to be the rule rather than the exception. That said, the current findings are consistent with results of other studies of parenting towards nontwin children using additional stressor variables [6], [7].

Sensitive and responsive parenting has been shown to be critical for children's cognitive and socioemotional development (e.g., [1]–[4], [59]–[61]). It is therefore important to continue the search for the factors that determine supportive as well as insensitive parent-child relationships. Greater understanding of these factors is needed to identify mother–child dyads at risk and to develop effective intervention programs. The current results indicate that the effects of child-rearing challenges on maternal sensitivity depend on mothers' genotype. The results portray the transactional processes between environmental risk factors and parents' genetic susceptibilities which generate individual differences in parents' abilities to sensitively interact with their children.
